# Moss establishment success is determined by the interaction between propagule size and species identity

**DOI:** 10.1038/s41598-022-24354-8

**Published:** 2022-12-01

**Authors:** Fernando Hurtado, Belén Estébanez, Pedro Aragón, Joaquín Hortal, Manuel Molina-Bustamante, Nagore G. Medina

**Affiliations:** 1 Department of Biogeography and Global Change, Museo Nacional de Ciencias Naturales (MNCN-CSIC), C/ Jose Gutierrez Abascal 2, 28006 Madrid, Spain; 2 Department of Biology (Botany), Universidad Autónoma de Madrid, 28049 Madrid, Spain; 3 Departament of Life Sciences, Universidad de Alcalá, 28805 Alcalá de Henares, Spain; 4 Centro de Investigación en Biodiversidad y Cambio Global (CIBC-UAM), Universidad Autónoma de Madrid, 28049 Madrid, Spain; 5 Department of Biodiversity, Ecology and Evolution, Faculty of Biological Sciences, Complutense University of Madrid (UCM), C/ José Antonio Novais, 12. Ciudad Universitaria, 28040 Madrid, Spain; 6 cE3c –Centre for Ecology, Evolution and Environmental Changes, Faculdade de Ciências da Universidade de Lisboa, 1749-016 Lisbon, Portugal

**Keywords:** Ecology, Plant sciences, Ecology

## Abstract

Colonization of new habitat patches is a key aspect of metacommunity dynamics, particularly for sessile organisms. Mosses can establish in new patches through fragmentation, with different vegetative structures acting as propagules. Despite the importance of these propagules for successful colonization the specific aspects that favour moss colonization by vegetative propagules remain poorly understood, including the effect of propagule size. We examine the intra- and interspecific variation of establishment and colonization success in culture of propagules of different sizes in six widespread soil moss species of contrasting growth form (*Dicranum scoparium*, *Homalothecium aureum*, *Hypnum cupressiforme*, *Ptychostomum capillare*, *Syntrichia ruralis* and *Tortella squarrosa*). We obtained three different size classes of propagules from artificially fragmented vegetative material, and assessed their establishment under controlled light and temperature conditions. We characterize the size, shape, apparent viability, morphological type and size changes due to hydration states of the propagules, all of them traits with potentially significant influence in their dispersal pattern and establishment. Then we assess the effect of these traits on moss establishment, using indicators of surface establishment (number of established shoots and colonized surface) and biomass production (viable biomass) as proxies of colonization success. The establishment indicators related to colonization surface and biomass production differ among species and propagule sizes. The magnitude of the interspecific differences of all indicators of establishment success was larger at the smaller propagule size class. *T. squarrosa* was the most successful species, and *D. scoparium* showed the lowest performance. We also found interspecific differences in the hydration dynamics of the propagules. The process of establishment by vegetative fragments operates differently among moss species. Besides, differences between hydration states in propagules of some species could be part of syndromes for both dispersal and establishment. This study unveils several functional traits relevant for moss colonization, such as wet *versus* dry area and length of fragments, which may improve our understanding of their spatial dynamics.

## Introduction

Understanding colonization dynamics is key for evolutionary biology and applied ecology as it modulates range shifts, connectivity between populations, and genetic fluxes^1,2^ Colonization is part of a multi-step invasion process that includes the generation of dispersible reproductive structures (propagules) in the source population, the dispersal of the propagules and their establishment^3,4^. Propagule production and dispersal are the first steps of the colonization process and imply a sufficient production of viable diaspores and its transport to a new locality^5^. However, the establishment is often the most limiting step for colonization^6–8^ (but see^4^), and thus determines metacommunity dynamics and changes in the limits of species distributions. It serves as the anchor of new dispersal events and the starting point of perdurable changes in both ecosystem and evolutionary dynamics. Indeed, establishing viable populations in new suitable habitat patches is determinant for species persistence under current global change scenarios (e.g.^9^). Due to this, there has been an increase in the number of studies that integrate migration potential into distribution modelling^10^. Nevertheless, for these approaches to be useful we need realistic data that account for an accurate estimation of the colonization potential. However, most studies analysing the effects of colonization processes on the ability of populations to track suitable environments focus mainly on the dispersal ability of the species, neglecting the role of their establishment potential and its links with diaspore traits.

Mosses are a good model for assessing establishment success experimentally. Like the rest of land plants, they are sessile organisms in which the establishment success depends directly on the ability to grow in situ. However, they have unique characteristics that make them interesting for the study of colonization processes. Although they reproduce by sexual spores (meiospores) or specialized vegetative propagules such as gemmae or bulbils, as in *Bryum* s.l., including *Ptychostomum*^11,12^, mosses are predominantly clonal organisms that routinely reproduce from undifferentiated vegetative tissues from non-specialized vegetative propagules^11,13–16^. Simple fragments of adult shoots, rhizoids and also detached leaves can serve as propagules, as in *Tortella squarrosa*^17^. This is so because in mosses even single cells have the potential to generate new plants (i.e. totipotency^18^). Fragmentation, as a vegetative reproduction mode, greatly increases the potential for local expansion of populations in the absence of, or complementary to, sexual reproduction^19^, and entails unique population dynamics among bryophytes^11,20–22^.

The size of the propagules is a key trait that can be important for the colonization process^23^. On the one hand, it has a significant influence in their dispersal^24^ mainly because larger propagules will be less likely to travel over long distances. However, large propagule sizes are not constrained solely to local colonization (e.g.^19,21,25,26^), and small propagule sizes can also be related mostly with local expansions^22,27,28^. Viable bryophyte fragments have been trapped from air with high abundances at long distances from their natural distribution area^19,29–31^, or in areas with very restricted occurrences^32^, and also attached to animals^33–36^ (see also a thorough revision in^37^), or even dispersed by humans^37,38^. Furthermore, relatively large moss cushion patches detached from the substrate can also act as colonization vehicles^39^. On the other hand, it is likely that the size of the vegetative propagules is important not only for dispersal but also for establishment success. This is a well-known trade-off for seed plants, where smaller seeds tend to have less potential of successful establishment. Smaller seeds have lower survival rates and emergence capabilities per seed, as they have less nutrient reserves than larger seeds^40^. Particularly, in bryophytes it is expected that larger propagules will have a higher establishment potential, as they may include a larger number of totipotent cells. This also happens between different types of propagules, as spores seem to have less viability than vegetative fragments^19,41^. However, there might be large differences among species, as not all species are equally successful in reproducing from leaves and shoots^11,19^. Besides, other factors could interact with size, although, for example, it is unknown if the presence and/or abundance of meristems on the vegetative propagules enhances their germination potential. In addition to size, propagule shape can also be important for establishment success. In vascular plants, relatively spherical dispersal structures (seeds, fruits, etc.) tend to live longer in the seed bank^42^. However, no previous study has directly addressed the effect of the shape of the propagules on their establishment success in mosses.

Despite the interest of the establishment stage for understanding plant colonization dynamics, the knowledge about establishment potential in bryophytes is scarce (but see^43^). Most studies analysing establishment in bryophytes approach it indirectly by studying invasiveness (e.g.^44^), or analysing particular contexts such as post-fire dynamics^45,46^ or restoration^47^. Only a few experimental studies have directly quantified moss establishment (e.g.^7,19,41,48^). Consequently, there is an important knowledge gap on the interspecific and intraspecific variability of establishment success in mosses. Thus, to better understand and predict the outcome of colonization processes and quantify their importance for evolutionary biology and applied ecology, we need to improve our knowledge on the intraspecific variability and the interspecific differences in the establishment potential of vegetative fragments.

In this work we used artificially fragmented vegetative propagules of six soil moss species to study: (1) the variation of their establishment success as a function of propagule size; (2) the influence of some morphological traits of the propagules; and (3) the importance of interspecific differences in the establishment success. We hypothesized that (1) larger propagule sizes will be associated to greater establishment success, and (2) the traits of the propagules will be determinant to explain differences in establishment success between species. Thus, we predicted that (1) propagules with more shoot tissue (potentially involving more meristematic apices) will establish better than leaf propagules, and (2) there will be interspecific differences in propagule culture success, favouring species where asexual reproduction is common, or even predominant. To these aims, we cultured artificially obtained fragments in the top surface of rockwool cubes to analyse their establishment and colonization success under controlled conditions, recording the number of established propagules in a given area, and the cover surface and biomass of the resulting moss patches after colonization. For each species, we characterized the range of sizes, projected area and morphological type of its propagules. Specifically, we studied *Dicranum scoparium* Hedw., *Homalothecium aureum* (Spruce) H. Rob., *Hypnum cupressiforme* Hedw., *Ptychostomum capillare* (Hedw.) D.T. Holyoak & N. Pedersen (= *Bryum capillare* Hedw.), *Syntrichia ruralis* F. Weber & D. Mohr and *Tortella squarrosa* (Brid.) Limpr. (= *Pleurochaete squarrosa* (Brid.) Lindb.) These species were selected because they are all soil species—which are usually easier to cultivate; have similarly broad distributions but co-occur in several regions; and are common in Europe, while showing different ecological and habitat preferences (e.g. types of soil, humidity gradient), and diverse adaptive growth strategies (e.g. mode of branching), thus providing insights on the functional diversification of moss responses.

## Material and methods

### Study species

The moss species studied have widespread distributions that span several continents^49,50^. All six species are common in the Iberian Peninsula, where they frequently coexist (e.g.^51–53^). They differ in ecological requirements regarding humidity (*D. scoparium* is hygrophyte-mesophyte, *H. cupressiforme* and *P. capillare* are mesophyte-xerophytes and *H. aureum*, *S. ruralis* and *T. squarrosa* are xerophytes) and soil type (e.g. *D. scoparium* and *P. capillare* are more humicolous, and *H. aureum* is more arenicolous) (see^49^). Besides, they also differ in their bearing of archegonia and growth form; *H. aureum* and *H. cupressiforme* are pleurocarpous mosses that grow flat to the ground, whereas *P. capillare, S. ruralis, T. squarrosa* and *D. scoparium* are acrocarpous species that grow normally erect. Additionally, *P. capillare* can develop specialized asexual propagules in rhizoids, stems and leaves^54^.

### Experimental design

The study consisted of two experiments, a pilot experiment to test different methods of artificial fragmentation and experimental conditions, and the main experiment, in which we tested the effects of propagule size and shape on establishment success.

In the pilot experiment we tested the six species, two different substrates (rockwool *vs*. felt), and four classes of fragments (fragments resulting from wet milling, and three size intervals of dry-milled fragments). We tested three replicates of all combinations (6 species × 2 substrates × 4 fragment milling treatments), thus we had a total sample size of 144. For wet milling, we first saturated the samples with water and milled them for 10 s in a regular electric coffee grinder with a single blade. Note that wet milling does not allow the separation of fragments of different size, and it may affect survival rates. For dry milling, we air-dried the samples and milled them in several successive steps. To separate the fragments by size we sieved the dry-milled propagules through meshes of different size (small: Ø 0.25–0.16 mm, medium: Ø 0.45–0.25 mm, large: Ø 0.75–0.63 mm). This method separates propagules by their maximum width but note that propagules can substantially vary in length, especially in the cases of long and narrow propagules. The propagules were sown in 2.5 × 2.5 cm seedling pots and kept in a growth chamber in controlled conditions (temperature of 10 °C, photoperiod 10 h light/14 h darkness, and a PAR radiation light of 31 µmol m^−2^ s^−1^), for 8 weeks. The results of the pilot experiment showed that there were no substantial differences between the establishment success of wet and dry fragments. Since dry milling allowed an easy separation of propagules by size, we used this milling method in the main experiment. Also, we used rockwool as substrate because it is an inert material that does not interfere with growth and required less watering. Before sowing the propagules, the rockwool was submerged in water, ensuring enough moisture for the propagules at initial stages. Additionally, to maintain humidity, the pots were placed over trays with a wet watering mat. After sowing, we watered the trays weekly to maintain humidity. This strategy maintained the surface of the substrate wet thanks to the high capillarity of rockwool, so no disturbance was applied to the culture when watering.

The main experiment consisted of a full factorial design including as factors the 6 species and three size classes of dry-milled fragments (small: Ø 0.25–0.16 mm, medium: Ø 0.45–0.25 mm, large: Ø 0.75–0.63 mm). We saved a portion of the dry-milled fragments of each size class and each species for the characterization of propagule traits. Total sample size for the main experiment was 216 (6 species × 3 fragment sizes × 12 replicates). Each sample consisted of 0.004 g (± 0.0002 g) of fragments sown in a 2.5 × 2.5 cm pot filled with rockwool. The experiment was done in the same culture conditions as the pilot experiment. The plants were allowed to grow for two months. At the end of the experiments, we made a qualitative assessment on the germination of the species observing whether the established shoots arose from leaf or shoot fragments.

We measured several parameters at the end of the experiment as indicators of establishment success: the number of established shoots, the percentage of surface covered by the established shoots in relation to the sample surface (colonized surface), the biomass of the established shoots (viable biomass) and non-viable biomass (non-established shoots and the rest of organic matter). Biomass was weighted after collecting and oven-drying the established shoots at 60ºC for 48 h. The biomass data (both viable and non-viable biomass, which account for final biomass) was used to estimate the percentage of viable biomass respect to the cultured biomass and the Relative Growth Rate (RGR), calculated as follows:

1 $${\text{Viable}}\;{\text{biomass}}\left( \% \right) = \left( {{\text{Viable}}\;{\text{biomass}}\left( {\text{g}} \right)/{\text{Cultured}}\;{\text{biomass}}\left( {\text{g}} \right)} \right) \times 100$$

2 $${\text{Relative}}\;{\text{Growth}}\;{\text{Rate}} = \left( {{\text{ln}}\;{\text{Final}}\;{\text{biomass}}\left( {\text{g}} \right) - \ln \;{\text{Cultured}}\;{\text{biomass}}\left( {\text{g}} \right)} \right)/{\text{Time}}\left( {{\text{month}}} \right)$$

These indicators account for local population growth as in Söderström and Herben^55^, taking into account that the shoots can be newly generated during the culture period, or be already present in the cultured propagules. This formula allows focusing only on newly generated biomass.

### Propagule trait characterization

To characterize the propagule traits, we measured 30 propagules under the optical microscope of each species and fragment size class (540 propagules in total) using samples saved from the main experiment. For each species and class size, the dry propagules were firstly put on a Petri dish, homogenizing the sample of the class size. To avoid biasing the selection of propagules we used a point sampling method that consisted of choosing the first 30 propagules following a line marked beforehand in the Petri dish.

Each propagule can be either a shoot or a leave fragment, according to the predominant tissue of the propagule. Shoot fragments are known for having greater germination potential than leaves mostly because they can contain meristematic apexes^19^. Thus, we counted 30 propagules per propagule size class and estimated the proportion of shoot propagules in the samples. Secondly, we measured a viability index (hereafter apparent viability) according to observable cell and tissue features including proportion of collapsed cells, cell colour and plastid integrity and abundance in the cells. We assigned to each propagule an ordinal value ranging from 1 (lowest apparent viability: a large number of collapsed or empty cells, most cells discoloured or brown-tinged, tissues appearing senescent) to 6 (highest apparent viability: few empty/collapsed cells, most cells with green, healthy-looking plastids) (for further information see Table S.1). The index was designed specifically for this experiment and is based on the previous experience of the team with bryophyte cultures. All records of apparent viability were done by the same researcher to ensure that the measurements were consistent. Lastly, we measured several quantitative size and shape traits in the same propagules in dry and hydrated states. For this purpose, we took photos of the same set of propagules, both dry and hydrated, and analysed the images using the software ImageJ^56^. For each propagule we measured area in mm^2^; maximum length in mm and circularity. Circularity was measured after Cox^57^ because it offers an easy-to-interpret and size-independent measure that varies between 0 and 1, 1 being a perfect circle. Additionally, we compared the area (Eq. 3) and maximum length (Eq. 4) of hydrated and dry propagules:

3 $${\text{Wet}}\;{\text{vs}}{.}\;{\text{dry}}\;{\text{area}} = \left( {{\text{Area}}\;{\text{of}}\;{\text{hydrated}}\;{\text{propagule}} - {\text{Area}}\;{\text{of}}\;{\text{dry}}\;{\text{propagule}}} \right)/{\text{Area}}\;{\text{of}}\;{\text{hydrated}}\;{\text{propagule}}$$

4 $${\text{Wet}}\;{\text{vs}}{.}\;{\text{dry}}\;{\text{length}} = \left( {{\text{Length}}\;{\text{of}}\;{\text{hydrated}}\;{\text{propagule}} - {\text{Length}}\;{\text{of}}\;{\text{dry}}\;{\text{propagule}}} \right)/{\text{Length}}\;{\text{of}}\;{\text{hydrated}}\;{\text{propagule}}$$

### Statistical analyses

To select the most relevant propagule traits we explored the correlations between the studied traits and the establishment success indicators using a correlogram and a network plot. The *p* values of the correlations were adjusted using post hoc Holm adjustment method.

We used analysis of deviance (Type III test) to determine the effects of (1) propagule size (classes large, medium and small), (2) species cultured (*D. scoparium*, *H. aureum*, *H. cupressiforme*, *P. capillare*, *S. ruralis* and *T. squarrosa*) and (3) the interaction between these two factors on establishment success. We measured establishment by using four indicators as dependent variables in the different analyses: number of established shoots, colonized surface, viable biomass and relative growth rate (RGR). We tested the deviations from normality and homoscedasticity of model residuals using Kolmogorov–Smirnov and Bartlett's tests for the main effects, and Levene's tests for the interactions. Since the tests showed large deviations from normality, we used robust models with IWLS (iteratively (re)weighted least squares) for all the indicators, and a box-cox transformation on number of established shoots, colonized surface and viable biomass, to cope with the violations of normality in these variables. Also, we used a type III analysis of variance with HC4 (heteroscedasticity-consistent) robust sandwich variance estimator to cope with heteroscedasticity for all the indicators. For RGR transformation was not needed, being enough with the robust model and robust sandwich estimator. Then, we performed planned contrasts for post hoc pairwise t-tests with Holm adjustment method for the *p*-values, to assess properly the interactions between the two factors. Finally, after adjusting the models we assessed that there was no overdispersion in the data. All statistical analyses and graphs were performed in R environment (version 4.1.2^58^). The statistical analyses and models were conducted using rstatix^59^, vcd^60^, dplyr^61^, car^62^, stats^58^ and robustbase^63^. The figures were done using ggpubr^64^, dplyr^61^, car^62^, stats^58^, psych^65^, corrr^66^, corrplot^67^, ggtext^68^, grid^58^, gridExtra^69^, cowplot^70^ and grDevices^58^. Further details of the packages used for each part and package versions can be found in the script available online (see Data availability statement).

### Plant collection statement

All specimens were identified by NGM, BE, FH and MM-B, and voucher specimens of all samples from the six species were deposited at MAUAM herbarium at the Universidad Autónoma de Madrid, Spain. All mosses were collected under permission of the Comunidad de Madrid, Dirección General de Medio Ambiente, reference 10/112725.9/19. All sampling locations are public lands, or public access-easements, so no permissions were required from land owners. All the plant experiments were in compliance with relevant institutional, national, and international guidelines and legislation.

## Results

### Establishment success

**Figure 1:**
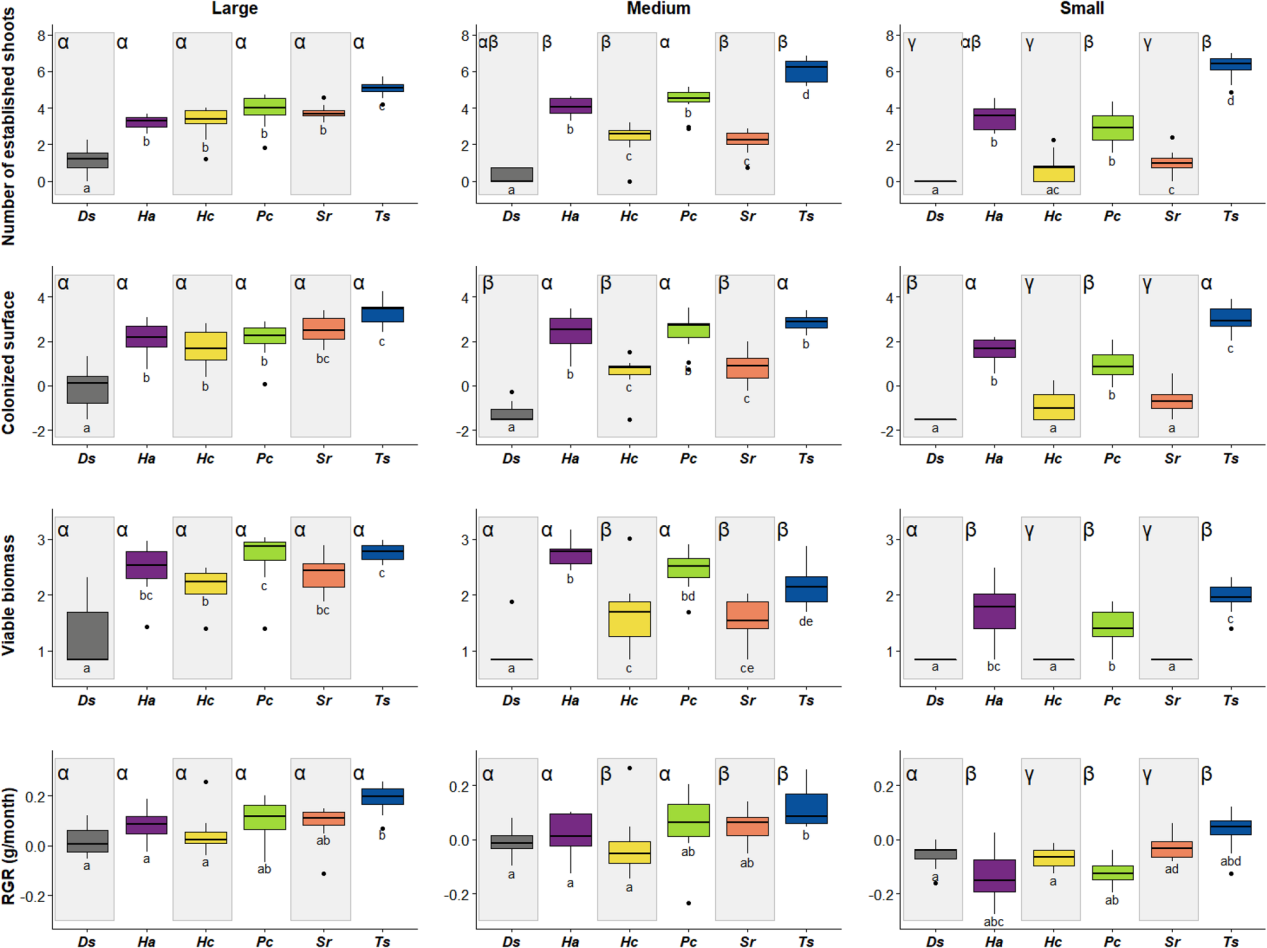
Boxplots of establishment success indicators for the analysed species (coloured) and propagule size classes (small, medium and large). Abbreviations: *Ds* for *D. scoparium, Ha* for *H. aureum, Hc* for *H. crupressiforme, Pc* for *P. capillare, Sr* for *S. ruralis* and *Ts* for *T. squarrosa.* Values of number of established shoots, colonized surface and viable biomass were Box–Cox transformed. Bars within each box represent the median, and boxes represent the interquartile range (IQR, top = 75th percentile, bottom = 25th percentile). Whiskers represent the largest/smallest value within 1.5 times IQR. Points represent outliers (> 1.5 times IQR). Compact letter display (CLD) shown as Latin (a, b, c, d and e) and greek letters (α, β and γ) represent, respectively: (1) significant differences across species within the same propagule size, (2) significant differences across propagule sizes within the same species. The values for CLD assignments are the *p*-adjusted values of pairwise comparisons for planned contrasts (Tables S2.1:S2.8).

All studied species were able to establish successfully from vegetative propagules. Even so, there were differences among the species in all indicators of successful establishment. Propagule size was a key factor regulating establishment success for all species. Larger propagule size classes tend to have greater establishment success (Fig. 1). However, there was a large interspecific variability in the establishment success of the propagules. The variability explained by the species, propagule size and their interaction was high for all the indicators of establishment success, but especially so for the number of established shoots and the colonized surface, where it was above 0.85 (Table 1).

**Table 1:** Results of the analysis of deviance testing the effect of species, propagule size and their interaction on the indicators of establishment success (number of established shoots, colonized surface, viable biomass and relative growth rate).

Response variable	Number of established shoots	Colonized surface	Viable biomass	Relative growth rate
**Treatments**				
Species	370.2	177.3	154.3	27.5
Propagule size	71.2	111.5	260.5	116.9
Species × propagule size	27.5	13.1	15.7	5.7
R^2^	0.90	0.85	0.79	0.59

R^2^ indicates the proportion of variance explained by the model including all three predictors. All parameters were significant at *p* < 0.001.

Overall, the magnitude of the interspecific differences of all indicators of establishment success was larger at the smaller propagule size class (Fig. 1). This implies that species differed largely in their ability to establish from small propagules. Also, the effect of propagule size varied with the indicator of establishment success. Regarding the number of established shoots, *D. scoparium* showed generally low numbers that decreased with propagule size, so that the smallest propagule size class presents the lowest number of established shoots in this species (Fig. 1). *H. cupressiforme* and *S. ruralis* showed an intermediate number of established shoots for the larger propagule size and a moderate decrease in the number of established shoots in the smallest propagule size. In contrast, *H. aureum* and *P. capillare* showed an intermediate number of established shoots that was similar in the three propagule size classes, while *T. squarrosa* showed high numbers of this indicator in all three propagule size classes. The response was very similar for the colonized surface (Fig. 1). However, the species showed a different pattern when considering the production of viable biomass and relative growth rate (RGR). For viable biomass, *D. scoparium* showed low values for all the propagule size classes (Fig. 1). While the rest of the species showed a decrease in the number of established shoots corresponding with smaller propagules, this decrease was sharper in *H. cupressiforme* and *S. ruralis* than in the rest of the species*.* Finally, RGR showed three different patterns (Fig. 1). Both *D. scoparium* and *H. cupressiforme* showed low RGR in all propagule size classes. In turn, *H. aureum*, *P. capillare* and *S. ruralis* showed a moderate decrease in RGR in the smaller propagule sizes. Finally, in *T. squarrosa* we observed a subtle decrease in RGR values with the decrease in propagule size and moderate RGR values in all propagule sizes. In general terms, there seemed to be no association between establishment success and growth forms traits or ecological requirements (see “Methods” and Fig. 1).

The six species studied showed heterogeneity in propagule traits (Table 2) and among propagule sizes (Tables S.3 and S.4). There was a very large interspecific variation in the percentage of shoots of the propagules. *P. capillare* showed the highest proportion of shoots, well above 80%. In the other end, *T. squarrosa* and *S. ruralis* showed the lowest proportion of shoots, below 25%. In contrast, the apparent viability of the propagules before culture did not show much variation among species. The average area of the propagules varied between the 0.69 mm^2^ of *P. capillare* and the 2.48 mm^2^ of *D. scoparium*. Similarly, there was a large amount of interspecific variability in the length of the propagules. *D. scoparium* had the longest propagules followed by *T. squarrosa* and *S. ruralis*. Concerning the shape, the species were more similar than in size traits. Most propagules in all six species were elongated in shape rather than circular, with circularity values between 0.32 and 0.48. Finally, all species showed a considerable increase in propagule area when the propagules were wet. This increase was maximum for the propagules of *S. ruralis* and *T. squarrosa*. The differences in length by hydration states were also subtle. When wet, the propagules of *D. scoparium*, *H. aureum* and *H. cupressiforme* did not elongate substantially, while the propagules of *P. capillare*, *S. ruralis* and *T. squarrosa* showed a moderate elongation.

**Table 2:** Summary of the average values and standard deviations of the size and shape traits for the six analysed species.

Species	Shoot %	Apparent viability	Area (mm^2^)	Length (mm)	Circularity	Wet versus dry area	Wet versus dry length
*D. scoparium*	52.2	5.16 (± 1.54)	2.48 (± 2.46)	3.08 (± 1.67)	0.34 (± 0.15)	0.31 (± 0.15)	0.06 (± 0.11)
*H. aureum*	51.1	5.39 (± 1.28)	1.14 (± 1.34)	1.90 (± 1.18)	0.33 (± 0.16)	0.35 (± 0.18)	0.07 (± 0.12)
*H. cupressiforme*	41.1	5.28 (± 1.41)	0.80 (± 0.88)	1.56 (± 0.79)	0.43 (± 0.15)	0.24 (± 0.30)	0.03 (± 0.22)
*P. capillare*	81.1	4.82 (± 1.61)	0.69 (± 0.53)	1.49 (± 0.66)	0.48 (± 0.17)	0.31 (± 0.17)	0.12 (± 0.09)
*S. ruralis*	17.7	5.44 (± 1.08)	1.46 (± 1.26)	2.39 (± 1.45)	0.46 (± 0.15)	0.48 (± 0.20)	0.14 (± 0.13)
*T. squarrosa*	23.3	5.62 (± 0.88)	1.12 (± 0.73)	2.42 (± 0.91)	0.32 (± 0.17)	0.54 (± 0.13)	0.34 (± 0.16)

**Figure 2:**
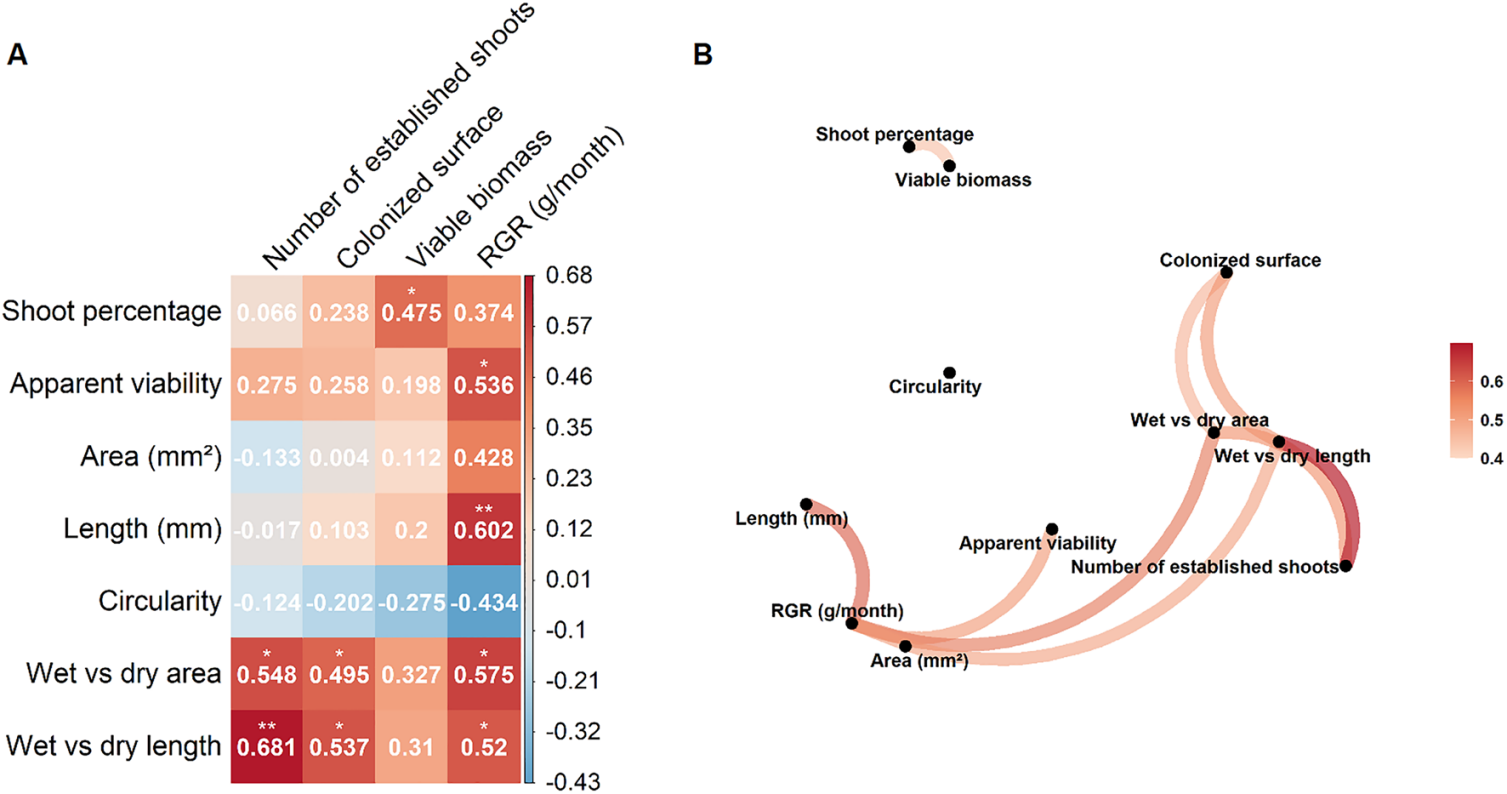
Correlations between the analysed traits and the indicators of establishment success. (**A**) Matrix of Pearson correlation coefficients. Correlations vary from red (+) to blue (−). Some indicators were previously transformed (see “Methods”). RGR stands for Relative Growth Rate. * *p* adjusted-values < 0.05, **p adjusted-values < 0.01. (**B**) Network plot for Pearson correlations. Links between points represent correlations. Only significant correlations are depicted (note that in this particular case all significant correlations are positive). Some indicators were previously transformed (see “Methods”). Indicators and traits are clustered according to their correlation coefficients: the proximity of the points are determined using multidimensional clustering. The appearing plot conditions for correlations are: (1) *p* adjusted-values < 0.05 and (2) *R* > 0.4. For the correlation values see A. For more detailed representations of the correlations shown, see Fig. S.1.

Some of these traits were significantly correlated (Figs. 2 and S.1), pointing to the existence of syndromes of associated traits (in this case, a group of traits related to a particular establishment strategy). Unsurprisingly, the highest correlations occurred between the size-related traits, and so, the propagules with larger area were also longer, and the propagules that increased more their area in wet also elongated more in wet conditions. There was a correlation between the shape and the size of the propagules, as larger propagules tended to be more elongated (lower circularity values). Interestingly, the proportion of shoots was larger in larger propagules classes (not shown).

In general, all establishment success indicators, except circularity and area, were positively correlated with propagule traits (Fig. 2). Relative Growth Rate (RGR) showed significant correlations with more traits than the rest of the establishment success indicators. The two traits related to the size increase of wet versus dry propagules showed the highest correlations with establishment success (relative growth rate, number of established propagules and colonized surface). The proportion of established shoots was the only trait correlated to the viable biomass.

## Discussion

Our results show that propagule size alone is not a good predictor of establishment success. For example, *Dicranum scoparium* had the largest propagule area and the lowest establishment success while *Ptychostomum capillare* had the smallest propagule area and a relatively high establishment success. These results imply the lack of a common pattern in the relationship between propagule size and establishment success along the vegetative propagules of the studied moss species. However, our experiments evidence that propagule size is critical for establishment success at the intraspecific level, as the smallest propagules had a reduced establishment success at least for one of the indicators in all species.

Many cells in the vegetative body of bryophytes are not irreversibly differentiated, and thus are able to produce a new plant^18^. Therefore, it could be argued that the larger a propagule is and the higher the number of cells it contains (even though cell size could vary between species), the higher the potential of successful establishment would be. However, this is only true if all the cells have a similar regenerative potential. Literature on the development from vegetative cells in bryophytes is sparse and for most species it is unknown whether the regeneration from any vegetative cell is possible or not (but see^17,71^). The few previous studies suggest the existence of large differences in the regeneration potential between species and plant tissues (e.g.^41^). For example, regeneration from leaves has been described only in a few species while regeneration from shoots seems to be more common (e.g.^19^). The lack of a consistent relationship between propagule size and establishment success confirms previous results. There seems to be a large degree of specialization in the ability of the species to reproduce from vegetative tissues. Our qualitative observations also point in this direction, and is in agreement with our presupposition of shoot propagules establishing better than leaf ones. We identified only two species that could routinely regenerate from leaves (*T. squarrosa* and *P. capillare*), while in the rest of the species regeneration occurred mostly from shoots. According to our indicators of establishment success, the most successful species is *T. squarrosa*, followed by *P. capillare*, in accordance with our expectations of higher success in the species that more commonly reproduce asexually^17,54,72^, with lesser effects of propagule size. Of course, the specific results we obtained are dependent on the environmental conditions of the experimental setting and it is possible that changing the temperature, humidity and/or light conditions would render different results. The changes in temperature might be the most influential as the species were grown in relatively cold temperatures (10 °C), so if the propagules of any of the species are sensitive to low temperatures it is possible that we underestimated their establishment potential. However, note that the three species that have more affinity for warmer temperatures (*Homalothecium aureum*, *Tortella squarrosa* and *Syntrichia ruralis*, see^49^) have a relatively high establishment success. So, this issue does not seem to have strongly affected our results. Besides, finding out that propagule success is dependent on temperature would only reinforce our main results that propagule size alone is not a good predictor of the establishment success because species specific differences are large and overcome the propagule size effect.

At the intraspecific level our results show that propagule size is the most relevant factor for explaining establishment success. Some species, such as *D. scoparium*, were unable to establish from very small propagules. Further, all species responded decreasing their biomass production and RGR as propagule size decreased. The very small propagules are more likely to disperse far from the source, so they are key for making a reliable assessment on the large-distance colonization potential of the species. Indeed, the analysed species showed two types of responses to propagule size. On the one hand, species in which propagule size had a null or small effect on the establishment success (*T. squarrosa* and to some extent also *H. aureum* and *P. capillare*); note also that *H. aureum* and *T. squarrosa* did not show such decreasing trend in colonized surface and number of established shoots. On the other, species in which propagule size had a strong negative effect on establishment success (*D. scoparium*, *Hypnum cupressiforme* and *Syntrichia ruralis*). Further refinement of this classification can be obtained by considering the overall establishment success, from *T. squarrosa*, the most successful species, to the very low success of *D. scoparium*. In fact, the low success of *D. scoparium* was striking and it might be related to the low resistance of the species to the fragmentation disturbance or to an absence of the necessary fungal communities in lab conditions (see^73^).

Interestingly, some of the analysed traits showed a positive association with establishment success. In particular, wet to dry area and propagule length showed the highest species-independent correlation with success. The mechanistic explanation for such importance of hydration is not straightforward but deserves further study because of its potential adaptive implications. The species with propagules that expand substantially in wet have relatively small propagules in dried states, which entails higher potential for long distance dispersal. At the same time these propagules will expand substantially in the wet conditions that are favourable for establishment, occupying more surface. In this experiment, larger differences between dry to wet states were found in *S. ruralis* and *T. squarrosa*, the two species that were able to reproduce extensively from leafy fragments. Interestingly, these two species also show high proportions of leafy fragments, suggesting that the species that produce a higher proportion of leafy fragments are also the ones that can reproduce from these small sized fragments^19^. This somehow contrasts with the common assumption about the importance of vegetative propagules for short distance dispersal. Vegetative fragments have been often associated to local maintenance or local expansions in the populations of mosses, while long distance dispersal^19^ has been associated to spores, which are smaller in size than propagules. However, our results show that at least some species can reproduce from very small fragments, suggesting that vegetative fragments can also play a significant role in long-distance dispersal. To ascertain this hypothesis future studies would need to analyse whether the small sized fragments can endure the stressful transport conditions^74–76^.

If our results are proved more general it would imply that propagule size alone cannot be used as a surrogate of establishment success. This, in turn, complicates the quantification of the importance of vegetative propagation for evolutionary biology and applied ecology applications. Furthermore, we found no significant association between establishment success and growth forms traits (pleurocarpous vs. acrocarpous) or taxonomic affinity. However, our study was not specifically designed to test hypotheses related to growth forms or taxonomic affinity thus their lack of importance needs further confirmation. In turn, we contribute to unveiling several relevant traits, such as shoot percentage, apparent viability, length and wet *versus* dry area and length, that in our experiments showed a positive association with establishment success and hence to individual fitness. Even so, it is also remarkable that while fragmentation is considered specially common among pleurocarpous mosses^11^, acrocarpous mosses in this study (*T. squarrosa* and *S. ruralis*) also exhibited a different proportion of types of propagules produced after fragmentation (except *P. capillare*, Tables 1 and S.4, most of them shoots). This led us to question whether the proportion of fragments of different nature produced after fragmentation is a particular characteristic of the species. Clarifying this is relevant due to the implications at all stages in colonization, as establishment success vary among propagule types^41^. For example, in some species leaves produce gemmae, propagules smaller than shoots, being able to dispersal across longer distances^11^ (also this study), while others do not. These basic differences may become an ecological filter with implications for metapopulation dynamics and community assembly, particularly during recolonization processes. Here, the higher establishment success of *T. squarrosa* may be providing this species with a competitive advantage, allowing it to monopolize certain environments (*sensu*^77^) in the Mediterranean region. In fact, the abundance and ease of sampling of this species has resulted in the proposal of using it in systematic biomonitoring studies in this region, where commonly used pleurocarpous species are not so readily available^78^. However, it is unclear whether the results found in the lab apply to field conditions, where differences in other environmental requirements in moss establishment can stand out such as humidity or microtopography^79^, especially considering all six species. Interspecific interactions among moss species also need to be studied as they coexist in nature and could affect their colonization success. Also, differences in ecological requirements (e.g. humidity and soil type) and growth form (acrocarpous or pleurocarpous) could play a role in field conditions. Further insights can also be gained by exploring different fragmentation methods, as our method does not reflect the variety of natural fragmentation conditions. Simulations of wind and animal-mediated fragmentations combined with different hydration conditions may help better understand the role of vegetative fragments for dispersal in natural conditions.

To summarize, our results show that the vegetative establishment potential in mosses responds to propagule size. However, propagule size alone is not enough to assess such potential due to the large interspecific variability. Several of the traits we analysed affect establishment potential, in combination with propagule size. These traits and indicators may be helpful towards fulfilling the need of complementary indicators to better assess nature conservation^80^.The combination of wet to dry area, percentage of shoots after fragmentation and apparent viability determine species syndromes that have not been described before, which we hypothesize may play an important role in the colonization of new habitats by soil mosses. Given the lack of a unique proxy for vegetative establishment success, and the large interspecific differences, further systematic measures that allow interspecific comparisons are needed to advance our knowledge. Future efforts should focus on a better characterization of moss propagules, creation of databases with propagule traits and exploring the link between laboratory experiments and field conditions.

## Supplementary Information

Supplementary Information 1.

## Data Availability

Datasets and R script used for the analysis, results and figures are available online under Creative Commons BY-NC-SA 4.0 International License: https://github.com/Fernando-Hurtado/DCR/tree/main/Establishment.
