# Supplementary material for: Moss establishment success is determined by the interaction between propagule size and species identity
Source: Sci Rep. 2022 Dec 1;12:20777. doi: 10.1038/s41598-022-24354-8 (PMC9715719; doi:10.1038/s41598-022-24354-8)
Supplement: Supplementary file 1 — Supplementary Information 1. [file 41598_2022_24354_MOESM1_ESM.docx]

Supporting information for Hurtado et al. Moss establishment success is determined by the interaction between propagule size and species identity.


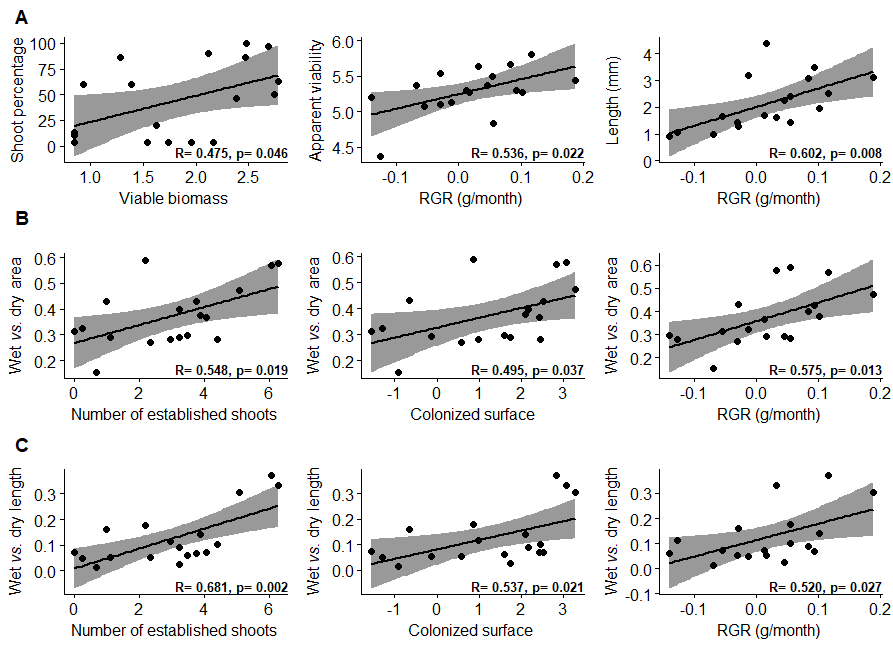


Figure S.1. Pearson correlations between establishment indicators and characterization traits. A: correlations of analysed traits that are correlated with only one establishment success indicator; B: correlations of establishment success indicators with wet *vs*. dry area *per* sample and C: similar to B but with wet *vs*. dry length *per* sample instead. Some indicators were transformed before correlations (see Methods). The appearing plot conditions of this figure are: 1) be significant (*p* adjusted values below 0.05) and 2) have R above 0.4. For the correlation values see Fig.2A.

| **Table S.1.** Apparent viability index | |
| --- | --- |
| Score | Description |
| 1 | Collapsed, discoloured or empty cells lacking plastids (>90%) |
| 2 | Collapsed cells (>80%) |
| 3 | Collapsed cells (60-80%) |
| 4 | Collapsed cells (40-60%), healthy-looking plastids in non-collapsed cells |
| 5 | Collapsed cells (20-40%), healthy-looking plastids in non-collapsed cells |
| 6 | Collapsed cells (<20%), healthy-looking plastids in non-collapsed cells |

| Table S.2.1 Number of established shoots, pairwise comparisons for planned contrasts (species *per* size). Levels of significance for the pairwise t tests (*p* adjusted by Holm) are: **** *p* ≠ 0, *** *p* < 0.0001, ** *p* < 0.001, * *p* < 0.01. | | | | |
| --- | --- | --- | --- | --- |
| Spp comparison | Size class | mean_dif | d | *p - value* |
| *D. scoparium* x *H. aureum* | Large | -1,64 | -3,84 | 1,13E-11 **** |
| *D. scoparium* x *H. cupressiforme* | Large | -1,68 | -2,86 | 3,21E-12 **** |
| *D. scoparium* x *P. capillare* | Large | -2,20 | -3,62 | 3,21E-19 **** |
| *D. scoparium* x *S. ruralis* | Large | -2,06 | -4,78 | 2,61E-17 **** |
| *D. scoparium* x *T. squarrosa* | Large | -3,16 | -7,21 | 2,75E-33 **** |
| *H. aureum* x *T. squarrosa* | Large | -1,52 | -4,77 | 3,44E-10 **** |
| *H. cupressiforme* x *T. squarrosa* | Large | -1,48 | -2,87 | 1,10E-09 **** |
| *P. capillare* x *T. squarrosa* | Large | -0,96 | -1,79 | 3,05E-04 *** |
| *S. ruralis* x *T. squarrosa* | Large | -1,10 | -3,40 | 1,61E-05 **** |
| *D. scoparium* x *H. aureum* | Medium | -2,99 | -9,45 | 9,40E-31 **** |
| *D. scoparium* x *H. cupressiforme* | Medium | -1,62 | -3,35 | 1,82E-11 **** |
| *D. scoparium* x *P. capillare* | Medium | -3,25 | -6,87 | 1,52E-34 **** |
| *D. scoparium* x *S. ruralis* | Medium | -1,50 | -4,05 | 6,72E-10 **** |
| *D. scoparium* x *T. squarrosa* | Medium | -4,62 | -11,0 | 6,66E-54 **** |
| *H. aureum* x *H. cupressiforme* | Medium | 1,37 | 2,65 | 2,22E-08 **** |
| *H. aureum* x *S. ruralis* | Medium | 1,49 | 3,64 | 7,16E-10 **** |
| *H. aureum* x *T. squarrosa* | Medium | -1,63 | -3,58 | 1,57E-11 **** |
| *H. cupressiforme* x *P. capillare* | Medium | -1,62 | -2,60 | 1,79E-11 **** |
| *H. cupressiforme* x *T. squarrosa* | Medium | -3,00 | -5,13 | 7,74E-31 **** |
| *P. capillare* x *S. ruralis* | Medium | 1,75 | 3,24 | 4,34E-13 **** |
| *P. capillare* x *T. squarrosa* | Medium | -1,37 | -2,39 | 1,98E-08 **** |
| *S. ruralis* x *T. squarrosa* | Medium | -3,12 | -6,32 | 1,03E-32 **** |
| *D. scoparium* x *H. aureum* | Small | -2,68 | -7,44 | 4,00E-26 **** |
| *D. scoparium* x *P. capillare* | Small | -2,27 | -4,62 | 2,84E-20 **** |
| *D. scoparium* x *S. ruralis* | Small | -0,75 | -2,03 | 1,32E-02 * |
| *D. scoparium* x *T. squarrosa* | Small | -5,01 | -12,2 | 5,20E-59 **** |
| *H. aureum* x *H. cupressiforme* | Small | 2,16 | 3,99 | 1,07E-18 **** |
| *H. aureum* x *S. ruralis* | Small | 1,92 | 3,72 | 2,23E-15 **** |
| *H. aureum* x *T. squarrosa* | Small | -2,33 | -4,28 | 4,20E-21 **** |
| *H. cupressiforme* x *P. capillare* | Small | -1,76 | -2,76 | 3,49E-13 **** |
| *H. cupressiforme* x *T. squarrosa* | Small | -4,50 | -7,80 | 3,08E-52 **** |
| *P. capillare* x *S. ruralis* | Small | 1,52 | 2,47 | 3,53E-10 **** |
| *P. capillare* x *T. squarrosa* | Small | -2,74 | -4,27 | 5,38E-27**** |
| *S. ruralis* x *T. squarrosa* | Small | -4,26 | -7,70 | 5,66E-49 **** |

| Table S.2.2 Number of established shoots, pairwise comparisons for planned contrasts (size classes). Levels of significance for the pairwise t tests (*p* adjusted by Holm) are: **** *p* ≠ 0, *** *p* < 0.0001, ** *p* < 0.001, * *p* < 0.01. | | | | |
| --- | --- | --- | --- | --- |
| Species | Size class comparison | mean_dif | d | *p - value* |
| *D. scoparium* | Large x Small | 0,836 | 2,26 | 3,28E-03 ** |
| *D. scoparium* | Medium x Small | -0,690 | -2,06 | 3,60E-02 * |
| *H. aureum* | Large x Medium | 0,720 | 1,12 | 2,24E-02* |
| *H. cupressiforme* | Large x Medium | 2,01 | 3,28 | 1,66E-16 **** |
| *H. cupressiforme* | Large x Small | 1,29 | 2,13 | 1,77E-07 **** |
| *H. cupressiforme* | Medium x Small | 0,763 | 1,11 | 1,14E-02 * |
| *P. capillare* | Large x Small | 1,15 | 1,75 | 4,75E-06 **** |
| *P. capillare* | Medium x Small | 1,23 | 3,15 | 7,82E-07 **** |
| *S. ruralis* | Large x Medium | 2,15 | 4,97 | 1,83E-18 **** |
| *S. ruralis* | Large x Small | 0,920 | 1,88 | 7,08E-04 *** |
| *S. ruralis* | Medium x Small | -0,798 | -1,80 | 6,20E-03 ** |
| *T. squarrosa* | Large x Medium | -1,01 | -2,14 | 1,11E-04 *** |
| *T. squarrosa* | Large x Small | 0,836 | 2,26 | 3,28E-03 ** |

| Table S.2.3 Colonized surface, pairwise comparisons for planned contrasts (species *per* size). Levels of significance for the pairwise t tests (*p* adjusted by Holm) are: **** *p* ≠ 0, *** *p* < 0.0001, ** *p* < 0.001, * *p* < 0.01. | | | | |
| --- | --- | --- | --- | --- |
| Spp comparison | Size class | mean_dif | d | *p - value* |
| *D. scoparium* x *H. aureum* | Large | -2,31 | -2,86 | 1,44E-13 **** |
| *D. scoparium* x *H. cupressiforme* | Large | -1,88 | -2,21 | 2,08E-09 **** |
| *D. scoparium* x *P. capillare* | Large | -2,24 | -2,65 | 6,73E-13 **** |
| *D. scoparium* x *S. ruralis* | Large | -2,65 | -3,42 | 3,25E-17 **** |
| *D. scoparium* x *T. squarrosa* | Large | -3,42 | -4,60 | 7,79E-26 **** |
| *H. aureum* x *T. squarrosa* | Large | -1,11 | -1,84 | 2,45E-03 ** |
| *H. cupressiforme* x *T. squarrosa* | Large | -1,54 | -2,32 | 2,14E-06 **** |
| *P. capillare* x *T. squarrosa* | Large | -1,18 | -1,80 | 9,32E-04 *** |
| *D. scoparium* x *H. aureum* | Medium | -3,72 | -5,88 | 2,93E-29 **** |
| *D. scoparium* x *H. cupressiforme* | Medium | -1,87 | -3,07 | 2,89E-09 **** |
| *D. scoparium* x *P. capillare* | Medium | -3,72 | -5,58 | 2,76E-29 **** |
| *D. scoparium* x *S. ruralis* | Medium | -2,14 | -3,71 | 7,61E-12 **** |
| *D. scoparium* x *T. squarrosa* | Medium | -4,11 | -10,5 | 8,96E-34 **** |
| *H. aureum* x *H. cupressiforme* | Medium | 1,85 | 2,42 | 4,05E-09 **** |
| *H. aureum* x *S. ruralis* | Medium | 1,58 | 2,13 | 9,10E-07 **** |
| *H. cupressiforme* x *P. capillare* | Medium | -1,85 | -2,33 | 3,87E-09 **** |
| *H. cupressiforme* x *T. squarrosa* | Medium | -2,24 | -3,84 | 6,32E-13 **** |
| *P. capillare* x *S. ruralis* | Medium | 1,58 | 2,06 | 8,86E-07 **** |
| *S. ruralis* x *T. squarrosa* | Medium | -1,97 | -3,58 | 2,83E-10 **** |
| *D. scoparium* x *H. aureum* | Small | -3,13 | -9,05 | 1,64E-22 **** |
| *D. scoparium* x *P. capillare* | Small | -2,51 | -5,26 | 9,74E-16 **** |
| *D. scoparium* x *T. squarrosa* | Small | -4,59 | -11,3 | 2,76E-39 **** |
| *H. aureum* x *H. cupressiforme* | Small | 2,50 | 4,31 | 1,46E-15 **** |
| *H. aureum* x *S. ruralis* | Small | 2,24 | 4,10 | 6,32E-13 **** |
| *H. aureum* x *T. squarrosa* | Small | -1,47 | -2,76 | 7,33E-06 **** |
| *H. cupressiforme* x *P. capillare* | Small | -1,88 | -2,83 | 2,06E-09 **** |
| *H. cupressiforme* x *T. squarrosa* | Small | -3,96 | -6,43 | 4,16E-32 **** |
| *P. capillare* x *S. ruralis* | Small | 1,63 | 2,55 | 3,64E-07 **** |
| *P. capillare* x *T. squarrosa* | Small | -2,08 | -3,32 | 2,61E-11 **** |
| *S. ruralis* x *T. squarrosa* | Small | -3,71 | -6,32 | 3,42E-29 **** |

| Table S.2.4 Colonized surface, pairwise comparisons for planned contrasts (size classes). Levels of significance for the pairwise t tests (*p* adjusted by Holm) are: **** *p* ≠ 0, *** *p* < 0.0001, ** *p* < 0.001, * *p* < 0.01. | | | | |
| --- | --- | --- | --- | --- |
| Species | Size class comparison | mean_dif | d | *p - value* |
| *D. scoparium* | Large x Medium | 1,14 | 1,60 | 1,54E-03 ** |
| *D. scoparium* | Large x Small | 1,40 | 2,17 | 2,32E-05 **** |
| *H. cupressiforme* | Large x Medium | 1,16 | 1,52 | 1,23E-03 ** |
| *H. cupressiforme* | Large x Small | 2,66 | 3,68 | 2,91E-17 **** |
| *H. cupressiforme* | Medium x Small | 1,49 | 2,13 | 4,47E-06 **** |
| *P. capillare* | Large x Small | 1,13 | 1,56 | 1,94E-03 ** |
| *P. capillare* | Medium x Small | 1,46 | 1,92 | 7,79E-06 **** |
| *S. ruralis* | Large x Medium | 1,66 | 2,55 | 2,03E-07 **** |
| *S. ruralis* | Large x Small | 3,17 | 5,26 | 5,44E-23 **** |
| *S. ruralis* | Medium x Small | 1,51 | 2,33 | 3,44E-06 **** |

| Table S.2.5 Viable biomass, pairwise comparisons for planned contrasts (species *per* size). Levels of significance for the pairwise t tests (*p* adjusted by Holm) are: **** *p* ≠ 0, *** *p* < 0.0001, ** *p* < 0.001, * *p* < 0.01. | | | | |
| --- | --- | --- | --- | --- |
| Spp comparison | Size class | mean_dif | d | *p - value* |
| *D. scoparium* x *H. aureum* | Large | -1,19 | -2,35 | 2,36E-12 **** |
| *D. scoparium* x *H. cupressiforme* | Large | -0,83 | -1,71 | 2,65E-06 **** |
| *D. scoparium* x *P. capillare* | Large | -1,40 | -2,70 | 1,74E-16 **** |
| *D. scoparium* x *S. ruralis* | Large | -1,10 | -2,43 | 9,01E-11 **** |
| *D. scoparium* x *T. squarrosa* | Large | -1,49 | -3,56 | 2,94E-18 **** |
| *H. cupressiforme* x *P. capillare* | Large | -0,57 | -1,36 | 5,98E-03 ** |
| *H. cupressiforme* x *T. squarrosa* | Large | -0,66 | -2,32 | 5,39E-04 *** |
| *D. scoparium* x *H. aureum* | Medium | -1,81 | -6,97 | 6,49E-25 **** |
| *D. scoparium* x *H. cupressiforme* | Medium | -0,69 | -1,44 | 2,13E-04 *** |
| *D. scoparium* x *P. capillare* | Medium | -1,54 | -4,88 | 3,02E-19 **** |
| *D. scoparium* x *S. ruralis* | Medium | -0,61 | -1,73 | 2,10E-03 ** |
| *D. scoparium* x *T. squarrosa* | Medium | -1,23 | -4,01 | 4,16E-13 **** |
| *H. aureum* x *H. cupressiforme* | Medium | 1,12 | 2,45 | 4,37E-11 **** |
| *H. aureum* x *S. ruralis* | Medium | 1,20 | 3,73 | 1,49E-12 **** |
| *H. aureum* x *T. squarrosa* | Medium | 0,58 | 2,17 | 4,69E-03 ** |
| *H. cupressiforme* x *P. capillare* | Medium | -0,85 | -1,73 | 1,48E-06 **** |
| *H. cupressiforme* x *T. squarrosa* | Medium | -0,54 | -1,11 | 1,40E-02 * |
| *P. capillare* x *S. ruralis* | Medium | 0,93 | 2,52 | 8,23E-08 **** |
| *S. ruralis* x *T. squarrosa* | Medium | -0,62 | -1,71 | 1,87E-03 ** |
| *D. scoparium* x *H. aureum* | Small | -0,89 | -2,90 | 2,94E-07 **** |
| *D. scoparium* x *P. capillare* | Small | -0,54 | -2,04 | 1,25E-02 * |
| *D. scoparium* x *T. squarrosa* | Small | -1,11 | -6,30 | 5,74E-11 **** |
| *H. aureum* x *H. cupressiforme* | Small | 0,89 | 2,90 | 2,94E-07 **** |
| *H. aureum* x *S. ruralis* | Small | 0,89 | 2,90 | 2,94E-07 **** |
| *H. cupressiforme* x *P. capillare* | Small | -0,54 | -2,04 | 1,25E-02 * |
| *H. cupressiforme* x *T. squarrosa* | Small | -1,11 | -6,30 | 5,74E-11 **** |
| *P. capillare* x *S. ruralis* | Small | 0,54 | 2,04 | 1,25E-02 * |
| *P. capillare* x *T. squarrosa* | Small | -0,57 | -1,78 | 6,38E-03 ** |
| *S. ruralis* x *T. squarrosa* | Small | -1,11 | -6,30 | 5,74E-11 **** |

| Table S.2.6 Viable biomass, pairwise comparisons for planned contrasts (size classes). Levels of significance for the pairwise t tests (*p* adjusted by Holm) are: **** *p* ≠ 0, *** *p* < 0.0001, ** *p* < 0.001, * *p* < 0.01. | | | | |
| --- | --- | --- | --- | --- |
| Species | Size class comparison | mean_dif | d | *p - value* |
| *H. aureum* | Large x Small | 0,74 | 1,71 | 5,73E-05 **** |
| *H. aureum* | Medium x Small | 1,00 | 2,93 | 4,31E-09 **** |
| *H. cupressiforme* | Large x Medium | 0,49 | 0,97 | 3,93E-02 * |
| *H. cupressiforme* | Large x Small | 1,27 | 4,80 | 6,64E-14 **** |
| *H. cupressiforme* | Medium x Small | 0,78 | 1,80 | 1,50E-05 **** |
| *P. capillare* | Large x Small | 1,30 | 3,09 | 1,77E-14 **** |
| *P. capillare* | Medium x Small | 1,08 | 3,06 | 1,90E-10 **** |
| *S. ruralis* | Large x Medium | 0,84 | 2,42 | 1,67E-06 **** |
| *S. ruralis* | Large x Small | 1,54 | 7,64 | 2,74E-19 **** |
| *S. ruralis* | Medium x Small | 0,70 | 2,46 | 1,83E-04 *** |
| *T. squarrosa* | Large x Medium | 0,62 | 2,51 | 1,87E-03 ** |
| *T. squarrosa* | Large x Small | 0,82 | 3,99 | 3,83E-06 **** |

| Table S.2.7 Relative Growth Rate, pairwise comparisons for planned contrasts (species *per* size). Levels of significance for the pairwise t tests (*p* adjusted by Holm) are: **** *p* ≠ 0, *** *p* < 0.0001, ** *p* < 0.001, * *p* < 0.01. | | | | |
| --- | --- | --- | --- | --- |
| Spp. comparison | Size class | mean_dif | d | *p - value* |
| *D. scoparium* x *T. squarrosa* | Large | -0,22 | -2,51 | 1,68E-06 **** |
| *H. aureum* x *T. squarrosa* | Large | -0,17 | -2,73 | 3,19E-02 * |
| *H. cupressiforme* x *T. squarrosa* | Large | -0,16 | -2,84 | 1,99E-04 *** |
| *D. scoparium* x *T. squarrosa* | Medium | 0,13 | 2,02 | 1,38E-03 ** |
| *H. aureum* x *T. squarrosa* | Medium | 0,07 | 0,95 | 3,72E-02 * |
| *H. cupressiforme* x *T. squarrosa* | Medium | 9,54E-04 | 0,01 | 9,77E-05 **** |
| *H. aureum* x *S. ruralis* | Small | -0,07 | -0,96 | 1,70E-02 * |
| *H. aureum* x *T. squarrosa* | Small | -0,02 | -0,33 | 1,62E-06 **** |
| *P. capillare* x *T. squarrosa* | Small | -0,22 | -4,33 | 1,64E-05 **** |

| Table S.2.8 Relative Growth Rate, pairwise comparisons for planned contrasts (size classes). Levels of significance for the pairwise t tests (*p* adjusted by Holm) are: **** *p* ≠ 0, *** *p* < 0.0001, ** *p* < 0.001, * *p* < 0.01. | | | | |
| --- | --- | --- | --- | --- |
| Species | Size class comparison | mean_dif | d | *p - value* |
| *H. aureum* | Large x Small | 0,05 | 0,94 | 8,05E-11 **** |
| *H. aureum* | Medium x Small | -0,17 | -3,12 | 3,89E-05 **** |
| *H. cupressiforme* | Large x Small | -0,07 | -1,05 | 1,01E-02 * |
| *P. capillare* | Large x Small | 0,04 | 0,45 | 2,64E-11 **** |
| *P. capillare* | Medium x Small | 0,08 | 1,32 | 2,71E-07 **** |
| *S. ruralis* | Large x Small | -0,08 | -0,96 | 3,50E-03 ** |
| *T. squarrosa* | Large x Small | 0,16 | 2,57 | 2,09E-05 **** |

| Table S.3 Wilcoxon test for wet *versus* dry area per propagule among species and propagule sizes. Levels of significance for the Wilcoxon tests (*p* adjusted by Holm) are: **** *p* ≠ 0, *** *p* < 0.0001, ** *p* < 0.001, * *p* < 0.01. | | | |
| --- | --- | --- | --- |
| Species | Propagule size | statistic | *p - value* |
| *D. scoparium* | Large | 10624 | 4,90E-02* |
| *H. cupressiforme* | Small | 11219 | 6,00E-03** |
| *P. capillare* | Medium | 10884 | 2,10E-02* |
| *S. ruralis* | Medium | 3269 | 6,77E-07**** |
| *T. squarrosa* | Large | 5524 | 4,40E-02* |
| *T. squarrosa* | Medium | 3275 | 6,77E-07**** |
| *T. squarrosa* | Small | 3301 | 7,39E-07**** |

| Table S.4 Pearson's Chi-squared test for the percentage of shoots after artificial fragmentation among species and propagule sizes. All tests were significant at *p* < 0.001. In the case of residuals, * and ** means the presence of residuals individually significant at approximately *α* = 0.05 and *α* = 0.0001 levels, respectively. | | | | |
| --- | --- | --- | --- | --- |
| Species | Residuals: large | Residuals: medium | Residuals: small | *p - value* |
| *D. scoparium* | 2,61 * | 0,590 | -3,20 * | 1,23E-08 |
| *H. aureum* | 3,75 * | -8,51E-02 | -3,66 * | 6,52E-13 |
| *H. cupressiforme* | 4,18 ** | -1,80 | -2,37 * | 1,96E-10 |
| *P. capillare* | 0,946 | 0,338 | -1,28 | 8,81E-04 |
| *S. ruralis* | 3,75 * | -1,88 | -1,88 | 2,64E-06 |
| *T. squarrosa* | 4,54 ** | -2,27 * | -2,27 * | 1,82E-09 |
